# Supplementary material for: Effect of Tetraphenylborate on Physicochemical Properties of Bovine Serum Albumin
Source: Molecules. 2021 Oct 29;26(21):6565. doi: 10.3390/molecules26216565 (PMC8588492; doi:10.3390/molecules26216565)

Supplementary Materials

Effect of Tetraphenylborate on Physicochemical Properties of Bovine Serum Albumin

Ola Grabowska [1,a], Małgorzata M. Kogut [1,a], Krzysztof Żamojć [1], Sergey A. Samsonov [1], Joanna Makowska [1], Aleksandra Tesmar [1], Katarzyna Chmur [1], Dariusz Wyrzykowski [1,*] and Lech Chmurzyński [1]

[1] Faculty of Chemistry; University of Gdańsk; Wita Stwosza 63; 80-308 Gdańsk; Poland; ola.grabowska@phdstud.ug.edu.pl (O.G.); malgorzata.kogut@phdstud.ug.edu.pl (M.M.K.); krzysztof.zamojc@ug.edu.pl (K.Ż.); sergey.samsonov@ug.edu.pl (S.S.); joanna.makowska@ug.edu.pl (J.M.); aleksandra.tesmar@ug.edu.pl (A.T.); k.chmur.820@studms.ug.edu.pl (K.C.); lech.chmurzynski@ug.edu.pl (L.C.)

[a] Authors contributed equally

[*] Correspondence: dariusz.wyrzykowski@ug.edu.pl

Supplementary Figure S1. RMSD of protein (upper panel) and $[B(Ph)_4]^-$ ions in site I (middle panels) and in site II (bottom panel) in the course of the MD simulation.

Supplementary Figure S2. LIE free energy of binding for $[B(Ph)_4]^-$ ions in site I (upper and middle panels) and in site II (bottom panel) in the course of the MD simulation.

Figure S1. RMSD of protein (upper panel) and $[B(Ph)_4]^-$ ions in site I (middle panels) and in site II (bottom panel) in the course of the MD simulation.

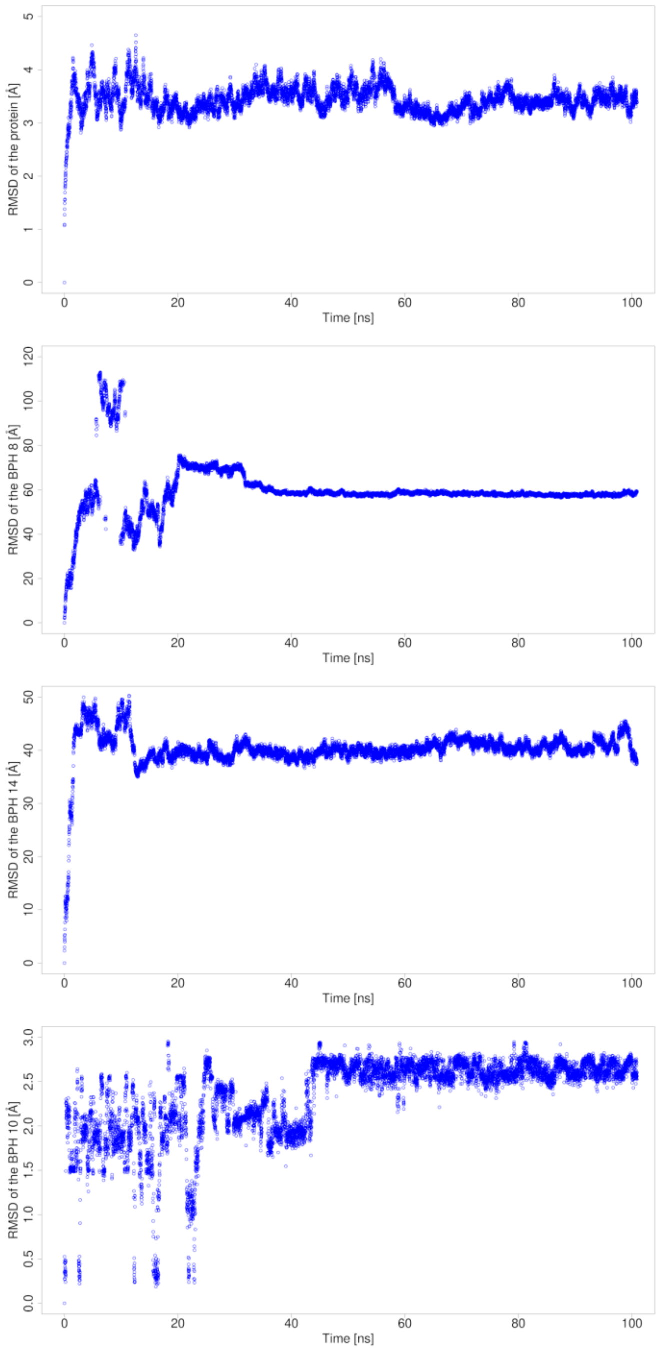

Figure S2. LIE free energy of binding for $[B(Ph)_4]^-$ ions in site I (upper and middle panels) and in site II (bottom panel) in the course of the MD simulation.

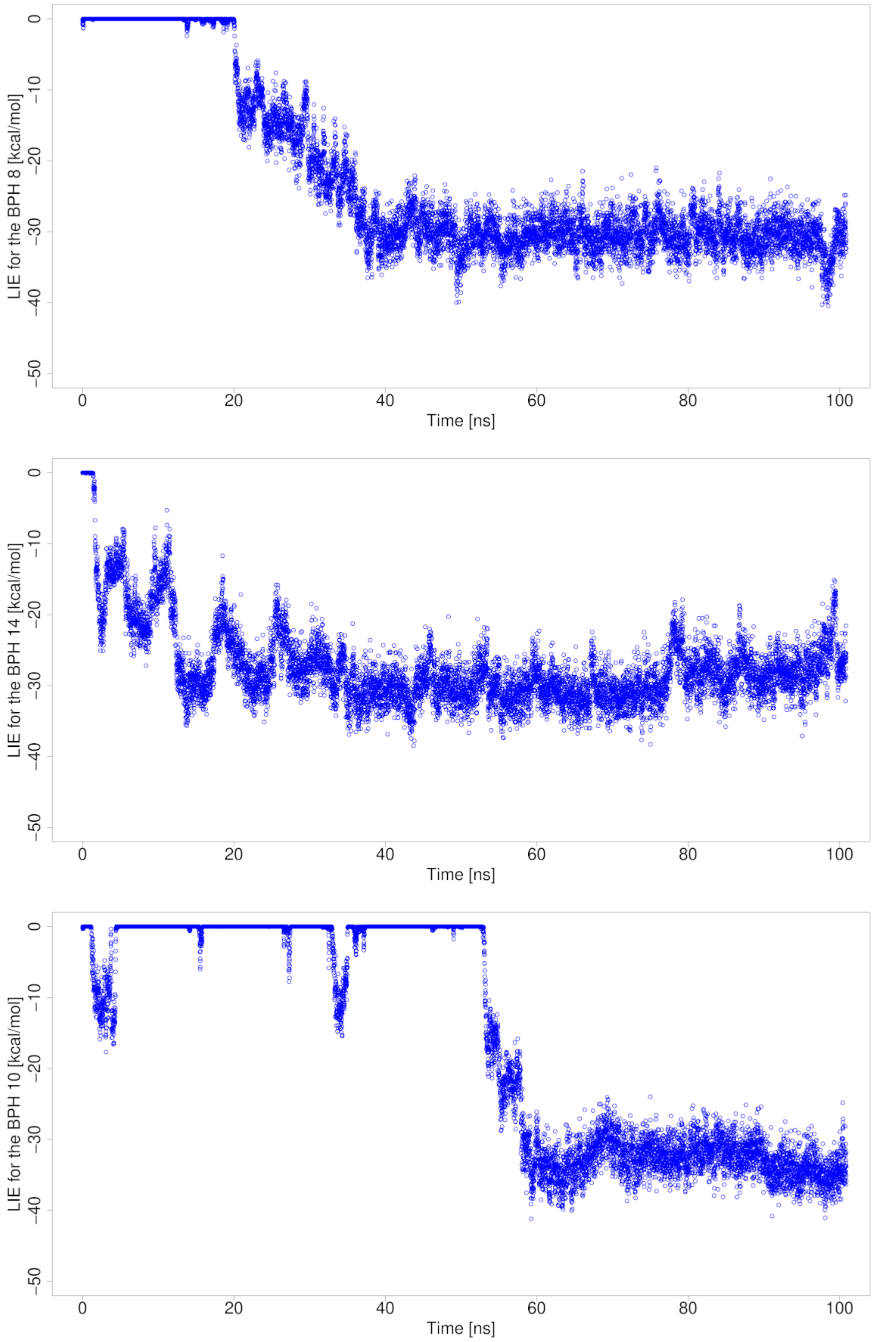

Supplement: Supplementary file 1 [file molecules-26-06565-s001.zip › molecules-1414570-supplementary.pdf]
